# Pulmonary infection by *Nocardia saintgeorgesii* mimicking lung cancer with concurrent pulmonary embolism in an immunocompetent host: a case highlighting the diagnostic role of mNGS

**DOI:** 10.3389/fcimb.2026.1765925

**Published:** 2026-02-25

**Authors:** Hang Hu, Dishan Cai, Juan Li, Kaijin Wang

**Affiliations:** 1 Bishan Hospital of Chongqing Medical University, Chongqing, China; 2 Chongqing Medical University, Chongqing, China; 3 Chongqing Liang Jiang New Area Traditional Chinese Medicine Hospital, Chongqing, China

**Keywords:** lung cancer misdiagnosis, mNGS, opportunistic bacterial infection, pulmonary embolism, pulmonary nocardiosis

## Abstract

**Background:**

Pulmonary nocardiosis presents a diagnostic challenge due to its frequent mimicry of lung cancer on imaging and the low sensitivity of conventional cultures. We report a case initially misdiagnosed as malignancy in an immunocompetent host, where metagenomic next-generation sequencing (mNGS) provided a definitive diagnosis and revealed a concurrent pulmonary embolism, suggesting a potential underrecognized association.

**Case presentation:**

This report describes a case of PN in an immunocompetent patient who was initially misdiagnosed with lung cancer based on imaging findings but later confirmed as pulmonary nocardiosis via mNGS. Notably, the patient also developed pulmonary embolism (PE). Empirical antibiotic therapy with piperacillin-tazobactam was initiated initially, supplemented with inhaled ipratropium bromide and expectorants to alleviate symptoms. Based on imaging findings suggestive of lung cancer, an invasive procedure was scheduled. mNGS was subsequently performed for further diagnosis. The subsequent results, along with CT scans, indicated no evidence of malignancy, leading to a consideration of Nocardia infection. The treatment regimen was then adjusted to ceftriaxone sodium combined with compound sulfamethoxazole, and the surgical schedule was canceled. The patient’s condition showed significant improvement, and he was discharged without fever or dyspnea. Some literature suggests that many PN patients present with concurrent deep vein thrombosis (DVT), suggesting a potential yet underrecognized association between Nocardia infection and thrombotic events. However, this correlation has not been fully reported before.

## Introduction

PN is an opportunistic infection primarily affecting immunocompromised individuals. However, recent years have seen increasing reports of PN in immunocompetent patients (Langmaid et al., 2021; Ye et al., 2023; Zhang et al., 2023; Bove et al., 2024; Daniel et al., 2024). Nocardia, a genus of gram-positive filamentous actinobacteria (Brown-Elliott et al., 2006), is widely distributed in soil and aquatic environments and can cause pulmonary, cutaneous, and disseminated infections, with pulmonary involvement being the most common (Wilson, 2012; Wang et al., 2022; Yang et al., 2023).

The radiological features of PN closely mimic those of pulmonary malignancies, posing significant diagnostic challenges. Typical imaging manifestations—such as nodules, cavitary lesions, or mass-like infiltrates (Restrepo and Clark, 2019; Thakur et al., 2020)—often lead to initial misdiagnosis as lung cancer, resulting in delayed treatment or unnecessary invasive procedures. Furthermore, the slow growth and low sensitivity of Nocardia in conventional cultures complicate definitive diagnosis, with many cases identified only at advanced stages (Liu et al., 2017), adversely impacting patient prognosis.

The advent of mNGS has revolutionized the early detection of rare and fastidious pathogens, offering a culture-independent, high-sensitivity diagnostic approach (Goldberg et al., 2015; Miao et al., 2018). Compared to traditional microbiological methods, mNGS enables broad-spectrum pathogen detection directly from clinical specimens (Simner et al., 2018), significantly improving diagnostic accuracy for PN and facilitating optimized individualized treatment strategies.

This study presents a case of PN in an immunocompetent patient initially misdiagnosed with lung cancer based on imaging findings but later confirmed by mNGS. Notably, the patient developed a concurrent PE, a complication that remains underrecognized in the context of PN. An increasing number of reported PN cases complicated by DVT suggest a potential association between Nocardia infection and thrombotic events. However, this phenomenon has yet to receive widespread attention, and relevant research remains limited.

This case underscores the importance of differentiating infectious from neoplastic pulmonary lesions, highlights the clinical utility of mNGS in PN diagnosis, and proposes a possible correlation between Nocardia infection and thrombosis warranting further investigation.

## Case presentation

### Patient information and initial symptoms

A 79-year-old male patient with a 5-year history of chronic obstructive pulmonary disease (COPD), well-controlled with regular inhalation of budesonide-formoterol, presented with a 10-day history of productive cough and exacerbation of exertional dyspnea following cold air exposure. He denied having fever, hemoptysis, chest pain, or a recent travel history prior to admission.

### Initial assessment and diagnostic workup

**Figure 1:**
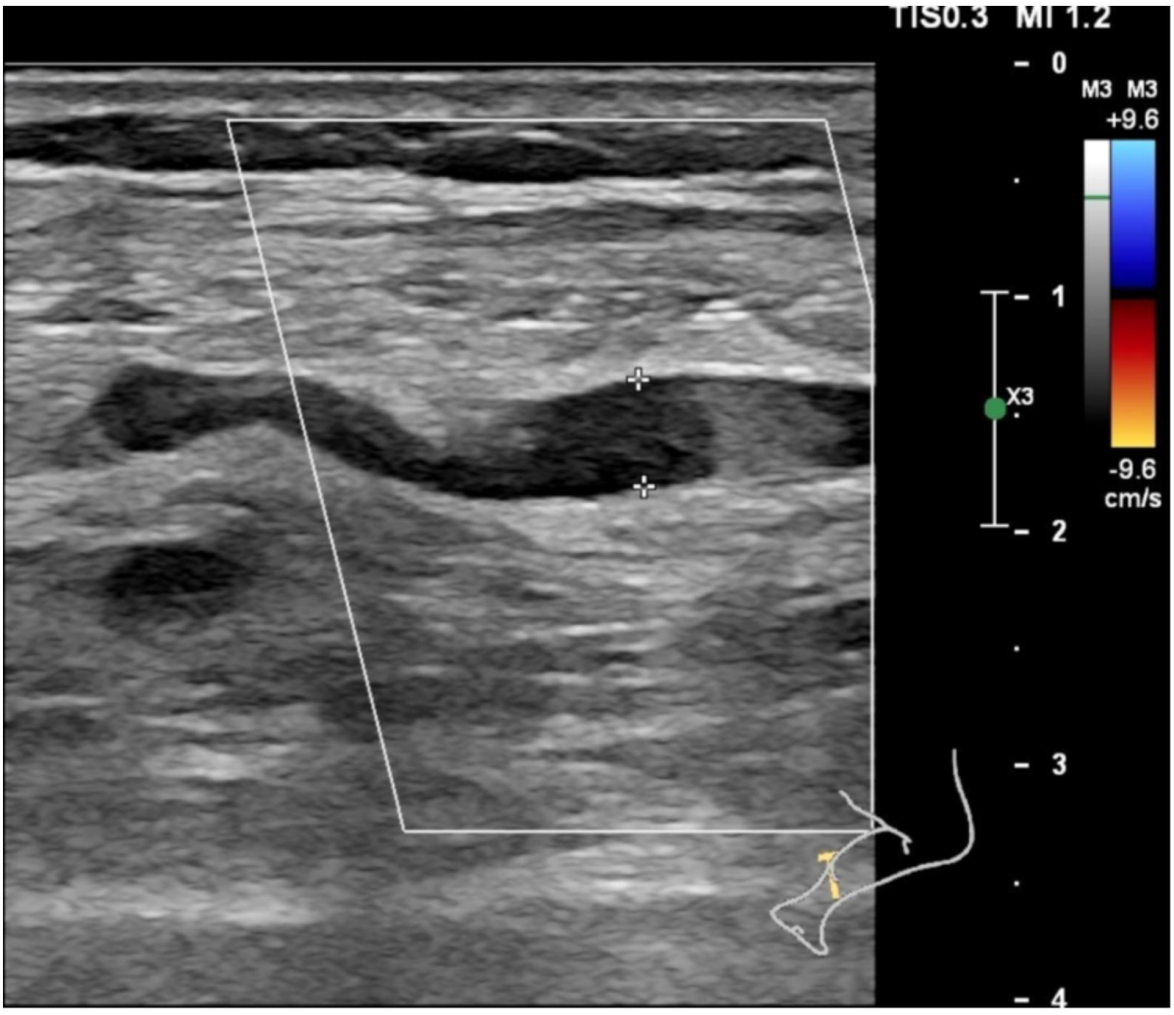
Lower extremity venous Doppler ultrasound revealed thrombosis in the intermuscular vein of the right calf.

On admission, his oxygen saturation was approximately 90% while receiving 2 L/min of supplemental oxygen. Laboratory tests revealed elevated inflammatory markers: D-dimer increased to 13.31 mg/L (normal range: 0.00–0.55 mg/L), fibrinogen rose to 8.40 g/L (normal range: 1.8–3.5 g/L), and IL-6 levels were markedly elevated to 227.50 pg/ml (normal range: 0–6.6 pg/ml). Additionally, total protein and serum albumin were significantly reduced (**Table 1**). The elevated CYFRA 21–1 level reported in **Table 1** may be attributed to ongoing alveolar epithelial injury and repair associated with the patient’s COPD (Heo et al., 2021). Bedside echocardiography showed normal ventricular systolic function, and Doppler ultrasound of the lower extremities detected an intermuscular venous thrombosis in the right calf (**Figure 1**).

**Table 1:** Laboratory.

Item	Actual value	Normal value
Complete blood count		
WBC	11.43 × 10^9^/L↑	(3.50–9.50) × 10^9^/L
Neutrophils	10.37 × 10^9^/L↑	(1.80–6.30) × 10^9^/L
Lymphocyte	0.74 × 10^9^/L	(1.10–3.20) × 10^9^/L
RBC	3.85× 10^12^/L	(4.30–5.80) × 10^12^/L
Hemoglobin	116 g/L	(130–175) g/L
Hematocrit	35.40%	(40.00–50.00)%
MCV	91.90fL	(82–100) fL
WBC	18.89 × 109/L↑	(3.50–9.50) ×109/L
Neutrophils	17.02 × 109/L↑	(1.80–6.30) × 109/L
Lymphocyte	0.82 × 109/L	(1.10–3.20) × 109/L
RBC	4.23× 1012/L	(4.30–5.80) × 109/L
Hemoglobin	104 g/L	(130–175) g/L
Platelet	426 ×109/L↑	(125–350) ×109/L
CRP	135.72 mg/L↑	(0–3) mg/L
PCT	0.308 ng/ml↑	(0–0.046) ng/mL
IL-6	227.50 pg/ml↑	(0–6.6) pg/mL
Biochemistry		
AST	30U/L	(12–37) U/L
ALT	17 U/L	(9–50) U/L
ALP	97 U/L	(45–125) U/L
GGT	14 U/L	(10–26) U/L
TBIL	20.2 umol/L	(0–26.0) umol/L
DBIL	6.0 umol/L	(0–8) umol/L
TP	66.20g/L	(65–85) g/L
Alb	35.10g/L	(40–55) g/L
Glb	31.10g/L	(20–40) g/L
BUN	4.63 mmol/L	(3.6–9.5) mmol/L
Creatinine	83 umol/L	(57–111) umol/L
eGFR	77.4mL/min	(60–120) mL/min
Tumor markers		
AFP	2.68ng/ml	<7.0ng/ml
CEA	1.67 ng/ml	<4.5ng/ml
CA19-9	5.29U/ml	<30U/ml
CYFRA 21-1	4.41ng/ml↑	<3.3ng/ml
SCC-Ag	0.67 ng/ml	(0-1.8)ng/ml
NSE	10.96 ng/ml	<16.5ng/ml
fPSA	0.13 ng/ml	<1.0ng/ml
tPSA	0.46 ng/ml	<4.0ng/ml
fPSA/tPSA Ratio	0.28	>0.16

**Figure 2:**
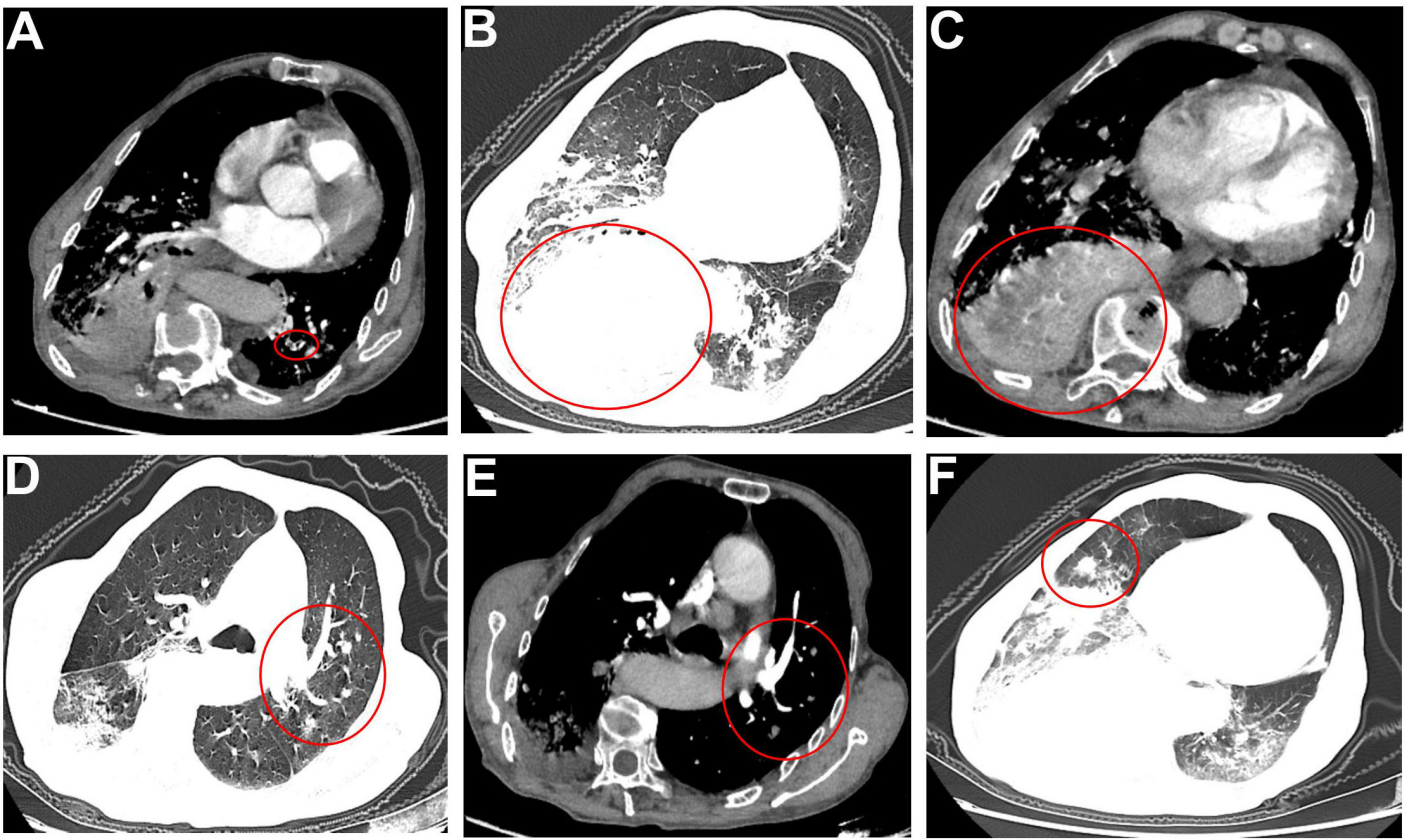
**(A)** A small patchy filling defect is observed in the posterior basal segment of the left lower lobe pulmonary artery, suggestive of pulmonary embolism. **(B, C)** Bronchial narrowing and occlusion are noted in the right lower lobe, accompanied by surrounding enlarged soft tissue with marked heterogeneous enhancement following contrast administration. These findings raise suspicion for a neoplastic lesion, along with obstructive pneumonia and atelectasis in the affected lobe. **(D–F)** Multiple nodules of varying sizes are scattered throughout both lungs. The lesions demonstrate heterogeneous enhancement on post-contrast images, with a high likelihood of pulmonary metastases.

Computed tomography pulmonary angiography (CTPA) revealed a small, patchy filling defect in the subsegmental pulmonary artery of the posterior basal segment of the left lower lobe. In the right lower lobe, bronchial narrowing and occlusion were identified, surrounded by irregular soft tissue with heterogeneous contrast enhancement, raising suspicion for a malignant process. Additionally, multiple nodules of varying sizes with heterogeneous enhancement were present in both lungs, suggesting the possibility of metastatic disease (**Figure 2**).

### Initial management and clinical course

The patient was hospitalized for a total of 17 days. Empirical antibiotic therapy with piperacillin–tazobactam was initiated on the first day of admission, accompanied by inhaled ipratropium bromide and expectorants to relieve symptoms. Given the diagnosis of pulmonary embolism, subcutaneous enoxaparin (4,000 IU every 12 hours) was administered. The patient’s cough and dyspnea improved, but intermittent low-grade fever developed after admission and has persisted since.

### Invasive evaluation and unexpected findings

**Figure 3:**
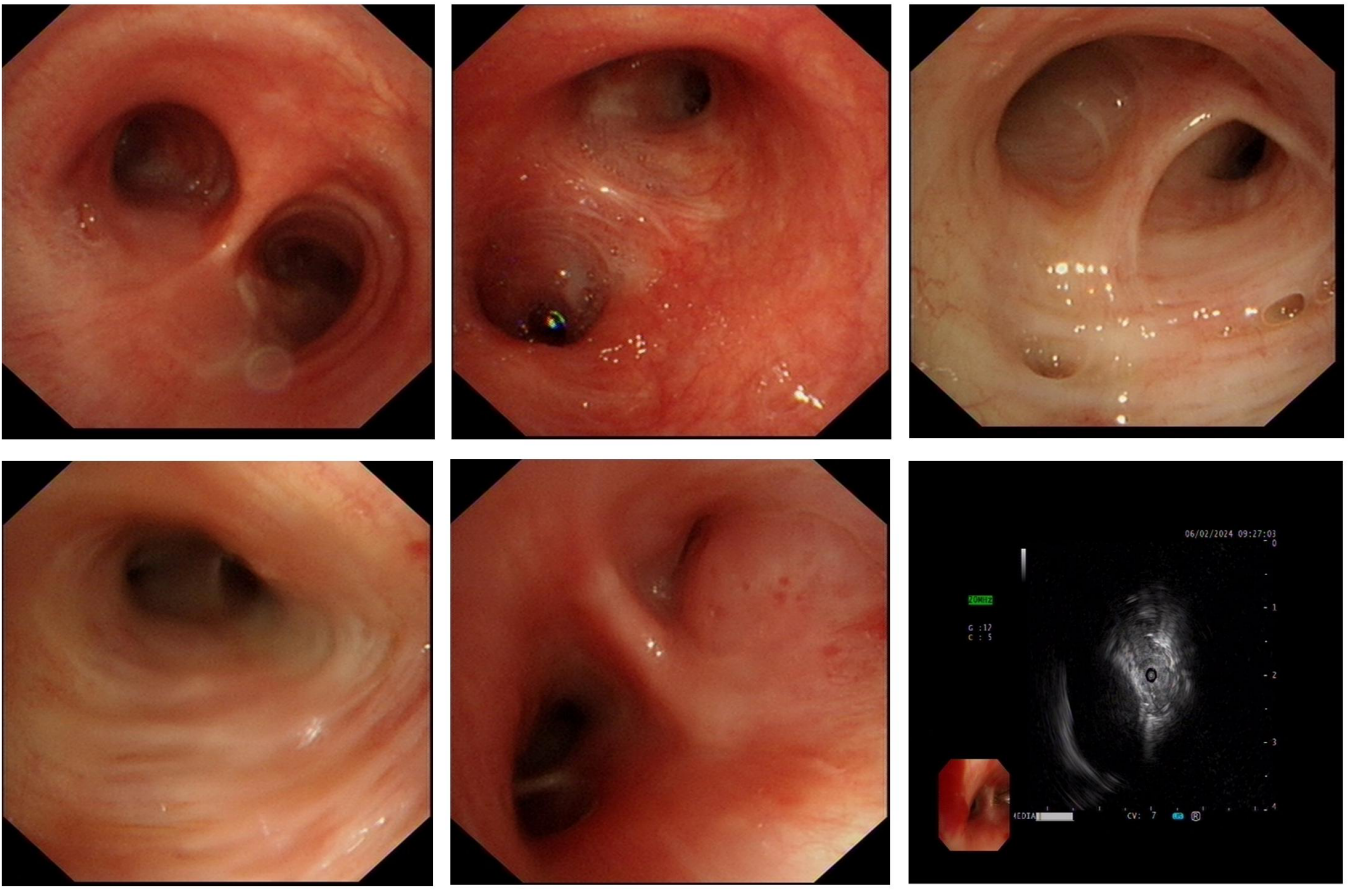
Bronchoscopy revealed extrinsic compression-induced narrowing of the right lower lobe bronchus. Bronchoalveolar lavage fluid (BALF) was collected for metagenomic next-generation sequencing (mNGS) analysis.

**Figure 4:**
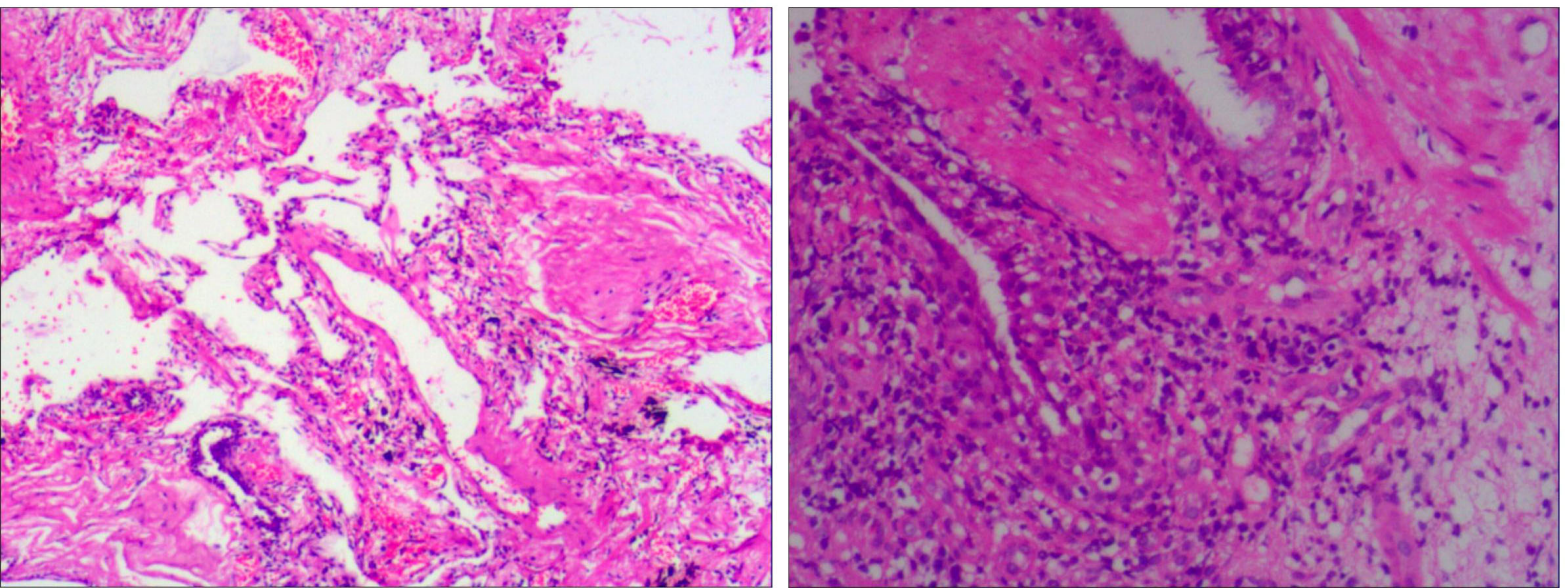
Histopathological examination revealed dense infiltration of inflammatory cells without evidence of malignant tumor cells.

To further clarify the diagnosis, flexible bronchoscopy was performed the day after admission, revealing extrinsic compression and luminal narrowing of the bronchus in the right lower lobe (**Figure 3**). At the same time, tissue samples obtained via transbronchial biopsy and bronchoalveolar lavage (BAL) were submitted for histopathological examination and mNGS, respectively. Histological analysis demonstrated abundant ciliated columnar epithelial cells with inflammatory infiltration, without evidence of malignancy (**Figure 4**).

A CT-guided percutaneous lung biopsy was subsequently scheduled. However, repeat imaging showed a marked reduction in the previously observed soft tissue density, a finding inconsistent with neoplastic pathology and more indicative of an infectious process. In light of these findings, the planned invasive procedure was cancelled.

### Pathogen identification and treatment response

**Figure 5:**
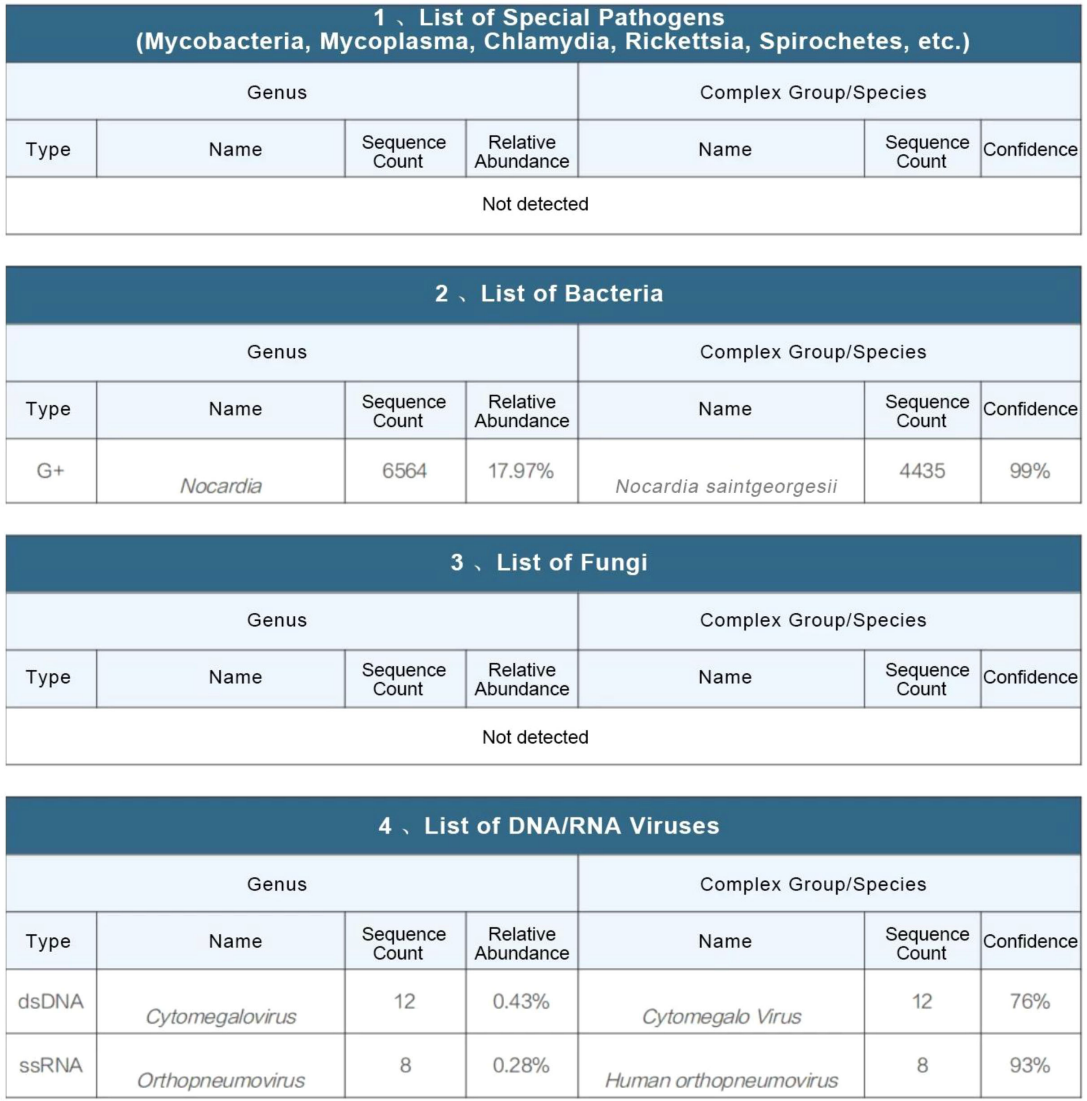
Original mMGS result image.

**Figure 6:**
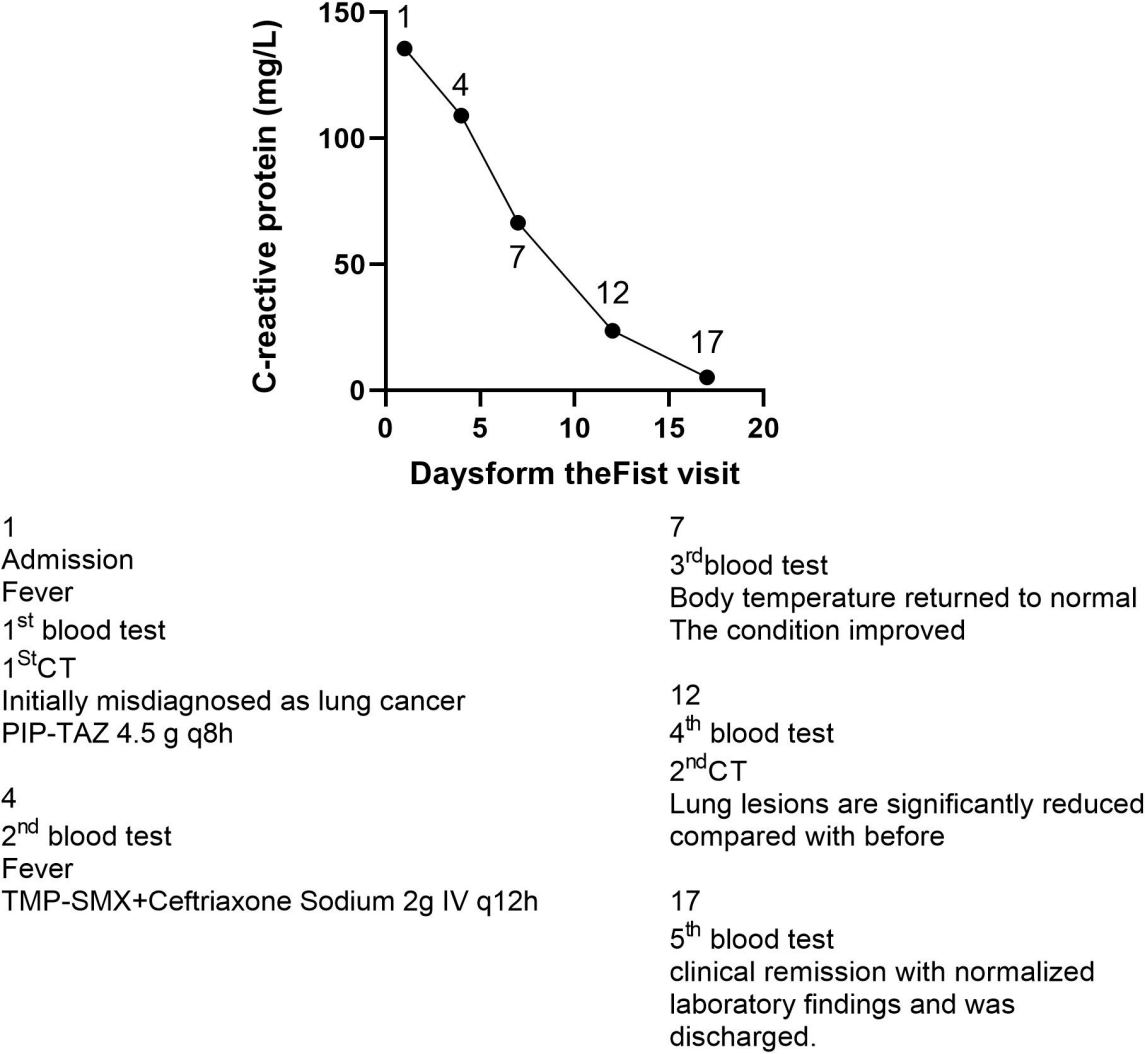
Changes in CRP levels after patient admission.

On the fourth day after admission, mNGS of the bronchoalveolar lavage fluid identified *Nocardia saintgeorgesii* (**Figure 5**), consistent with the patient’s clinical presentation. Simultaneously, sputum culture (including routine bacterial culture and Haemophilus influenzae culture) was performed over two days, with no pathogenic bacteria growth observed in the routine culture and negative results for Haemophilus influenzae culture. Based on this finding, the antimicrobial regimen was adjusted to ceftriaxone in combination with oral trimethoprim-sulfamethoxazole. The patient showed gradual clinical improvement, with marked relief of respiratory symptoms including cough, sputum production, and dyspnea (**Figure 6**).

**Figure 7:**
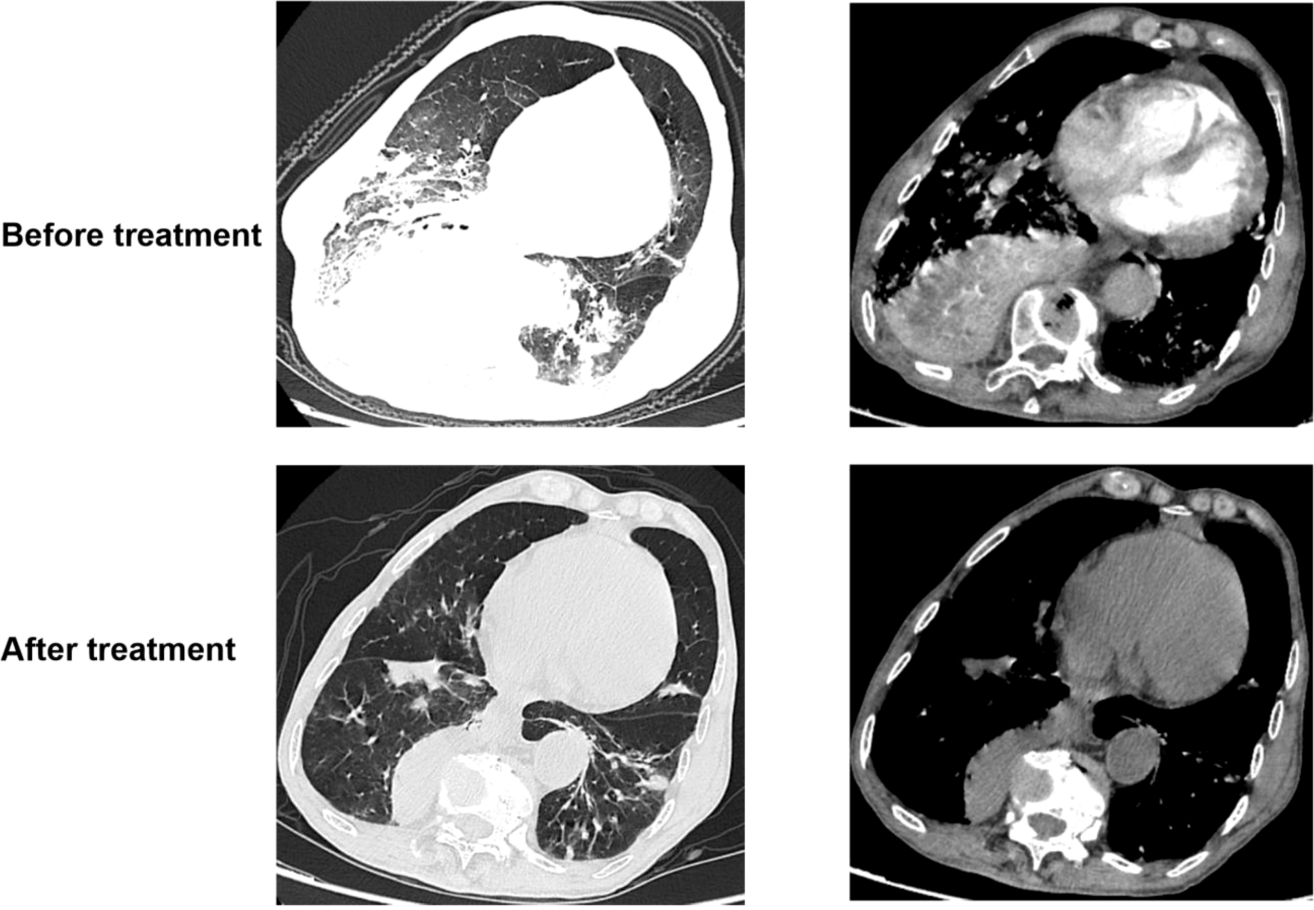
Follow-up chest CT revealed significant regression of the pulmonary lesions compared to the previous scan.

Follow-up laboratory tests two week later demonstrated significant improvement: white blood cell count was 3.39 × 109/L, neutrophil count 1.96 × 10^9^/L (58%), C-reactive protein (CRP) decreased to 5.16 mg/L, procalcitonin (PCT) to 0.042 ng/mL, and interleukin-6 (IL-6) to 12.79 pg/mL. Chest computed tomography (CT) revealed substantial resolution of the pulmonary lesions (**Figure 7**).

At the time of discharge, the patient was afebrile, breathing comfortably, and ambulating without signs of respiratory distress, indicating a stable clinical condition. A follow-up plan was arranged to monitor ongoing recovery and evaluate long-term treatment outcomes.

## Discussion

### Diagnostic challenges and the role of mNGS

PN remains a diagnostic challenge due to its nonspecific clinical manifestations. In particular, cases presenting with cavitary lesions or mass-like consolidations are frequently misdiagnosed as lung cancer (Restrepo and Clark, 2019; Thakur et al., 2020). In this case, chest imaging showed signs that strongly resembled malignancy, including bronchial obstruction, thickening of the tissue surrounding the bronchi, and multiple nodules with uneven contrast enhancement. These findings initially led clinicians to suspect lung cancer. However, standard diagnostic procedures—such as sputum cultures and bronchoscopic biopsies—provided relatively limited information. Since Nocardia species grow slowly and require specific culture conditions, they are sometimes missed by routine microbiological tests. In this patient, histopathological analysis of the bronchoscopic biopsy revealed only chronic inflammation, with no evidence of cancer or identifiable pathogens. This inconclusive result created a diagnostic inconvenience and led to consideration of a more invasive percutaneous biopsy for further clarification.

mNGS has emerged as a transformative tool for etiological diagnosis in infectious diseases. The broad-spectrum detection capability of mNGS allows for comprehensive pathogen identification, which is particularly beneficial in cases involving fastidious anaerobes, patients who have received empirical antibiotic therapy prior to sample collection, or complex infections where conventional tests fail to identify the causative agent (Chiu and Miller, 2019).

To identify the causative pathogen, metagenomic next-generation sequencing (mNGS) was performed on the bronchoalveolar lavage fluid using the Miniseq platform. The analysis exclusively detected *Nocardia saintgeorgesii* (sequence count: 6564), cytomegalovirus (sequence count: 12), and human orthopneumovirus (sequence count: 8) (**Figure 5**). The MetaCAP targeted pathogen sequencing technology can specifically capture 1,292 bacterial species, 517 fungal species, 1,380 viral species, 210 parasitic species, 2,627 resistance subtypes corresponding to 86 antimicrobial resistance genes, and 74 virulence genes. In the report, *Nocardia saintgeorgesii* was listed in the primary table with a high sequence count of 6564 RPM and a confidence level of 99%. Combined with its high relative abundance (accounting for 17.97% of bacterial sequences), it was identified as the primary pathogenic bacterium.

In this patient, mNGS analysis of BALF rapidly identified *Nocardia saintgeorgesii* as the causative pathogen. Combined with clinical correlation, this confirmed the diagnosis of PN, enabling prompt initiation of ceftriaxone and TMP-SMX therapy. The patient subsequently achieved significant symptomatic improvement and successful discharge.

### Pulmonary nocardiosis in immunocompetent hosts: an emerging concern

Traditionally considered an opportunistic infection (Wilson, 2012; Wang et al., 2022), PN primarily affects individuals with impaired cell-mediated immunity, including those receiving long-term corticosteroid therapy, solid organ transplant recipients, and chemotherapy patients. However, emerging evidence demonstrates increasing case reports among immunocompetent hosts (Langmaid et al., 2021; Ye et al., 2023; Zhang et al., 2023; Bove et al., 2024; Daniel et al., 2024). In a review of 1,000 cases of nocardiosis over 40 years, 38% of patients studied were neither immunocompromised nor had a risk factor (Duggal and Chugh, 2020).

Chronic structural lung diseases, including COPD and bronchiectasis, are now recognized as significant risk factors for PN, even in the absence of systemic immunodeficiency (Aggarwal et al., 2015; Steinbrink et al., 2018; Anand et al., 2025). These conditions disrupt local mucosal defenses, impair mucociliary clearance, and alter the pulmonary microbiota, creating a microenvironment conducive to opportunistic infections (Sze et al., 2014; Richardson et al., 2019). Additionally, subclinical immune dysfunction—such as impaired neutrophil function or defective alveolar macrophage activity—may compromise innate host defenses, particularly in elderly or chronically ill individuals (Berenson et al., 2013; Effah et al., 2021). Environmental exposure to Nocardia-contaminated soil or water, whether through occupational or regional contact, may also contribute to disease development in otherwise healthy hosts (Lam et al., 2022).

Notably, certain Nocardia species possess enhanced virulence factors that may contribute to rapid disease progression and increased mortality (Liu et al., 2017; Ott et al., 2019; Lepe-Balsalobre et al., 2021). These observations underscore the importance of maintaining a high index of clinical suspicion for nocardiosis, regardless of the patient’s immune status.

### A potential link between nocardia infection and thromboembolic events

A growing body of literature has reported an unexpectedly high incidence of DVT in patients with PN (Liu et al., 2017; Viscuse and Mohabbat, 2019; Douedi et al., 2020; Raslan et al., 2021; Pérez Ramos et al., 2024), suggesting a possible yet underappreciated link between Nocardia infection and thrombotic complications. In this case, the patient was found to have DVT during the first examination after admission, with no prior history of DVT or symptoms suggestive of venous thromboembolism before hospitalization. The patient regularly engaged in physical labor, had no history of malignancy, autoimmune diseases, trauma, recent surgery, or personal or family history of thromboembolic events. Although the patient had COPD, it was well controlled with regular medication. The concurrent occurrence of pulmonary embolism in the absence of traditional risk factors increases the possibility that the Nocardia infection itself may have created a prothrombotic environment.

While direct causal evidence remains lacking, the proposed link between Nocardia infection and thrombosis is biologically plausible. It is well documented that systemic infections can induce a prothrombotic state through complex crosstalk between inflammatory and coagulation pathways. As observed in other infectious diseases such as tuberculosis, Nocardia infection may similarly promote endothelial activation, upregulation of tissue factor, and release of pro-inflammatory cytokines including IL-6 and tumor necrosis factor-alpha (TNF-α), ultimately enhancing thrombin generation and suppressing fibrinolysis (Solis-Soto et al., 2008; Rosas-Taraco et al., 2012; Trevino-Villarreal et al., 2012).

In this case, the markedly elevated levels of inflammatory markers—CRP, PCT, and IL-6—were indicative of a strong systemic inflammatory response, which may have contributed to a procoagulant milieu and the development of pulmonary embolism.

Furthermore, the possibility of direct vascular involvement by Nocardia organisms cannot be entirely ruled out. Although uncommon, several reports have described vascular complications in disseminated nocardiosis, including cerebral vasculitis, retinal vasculopathy, and vascular invasion in soft tissues (Cheung et al., 1994; Ravage and Singerman, 2009; Lally et al., 2014). These observations raise the possibility that Nocardia may exhibit a degree of vascular tropism in certain hosts or under specific immunological conditions. The typical pathological manifestation of Nocardia infection is chronic suppurative granulomatous inflammation. Chronic granulomatous infectious foci serve as persistent and intense local inflammatory stimuli, representing one of the primary drivers of endothelial dysfunction. The markedly elevated inflammatory markers such as IL-6 and CRP in the patient act as key mediators that drive endothelial cell activation, compromise vascular integrity, and create a procoagulant microenvironment (Szmitko et al., 2003). Ultimately, the activated and dysfunctional endothelial cells lose their natural antithrombotic properties. Instead, they begin to express procoagulant substances such as tissue factor, promote the adhesion of platelets and leukocytes, and actively establish a local microenvironment conducive to thrombus formation (Prakash et al., 2022; Donadini et al., 2025). While the mechanism remains speculative, such endothelial or vascular wall involvement could locally disrupt vascular integrity, promote endothelial activation, and create a microenvironment conducive to thrombosis. In the context of pulmonary involvement, this may potentially contribute to *in situ* thrombus formation or exacerbate existing prothrombotic processes.

Given emerging evidence and biological plausibility, clinicians should maintain a high index of suspicion for thromboembolic events in patients with PN, particularly those exhibiting elevated D-dimer levels or unexplained respiratory deterioration. Further studies are warranted to elucidate the prevalence, pathophysiological mechanisms, and prognostic implications of thrombosis in the setting of Nocardia infection (Fu et al., 2017).

### Limitations of this case in clinical practice

Although a connection between Nocardia infection and thromboembolic complications seems possible, the study has clear limitations. Most notably, we lack direct proof of Nocardia in the thrombotic tissue and do not have serial data on relevant coagulation and fibrinolysis markers. This is indeed the major constraint for clinical interpretation. Still, as noted earlier, localized inflammation from Nocardia could promote endothelial damage. In this case, the activated endothelium, alongside the patient’s elevated inflammatory markers, might have established conditions favorable to clotting. Thus, we believe Nocardia could have played a role in thrombus development.

Though direct microscopy is a traditional method for the rapid diagnosis of nocardiosis, in this case, lung cancer was initially highly suspected, so the diagnostic focus was first placed on histopathological evaluation. Upon admission, we ran routine tests such as a sputum culture and sputum smear before starting antibiotic treatment. Due to elevated inflammatory markers and the need to consider infectious etiologies in the differential diagnosis, mNGS was also conducted. Since the sputum smear result was negative and malignancy was highly suspected at the time, infectious diseases were not prioritized in the diagnostic workup. Therefore, direct microscopic examination was not performed, which indeed reflects an oversight in our clinical consideration.

### Treatment considerations and clinical outcome

The patient initially received empirical antibiotic therapy with piperacillin–tazobactam. Although CRP levels showed a downward trend, low-grade fever persisted. Following the identification of Nocardia species, the regimen was switched to ceftriaxone combined with trimethoprim–sulfamethoxazole (TMP–SMX), leading to rapid defervescence and marked clinical improvement. TMP–SMX remains the cornerstone of Nocardia treatment due to its broad-spectrum activity against most species. In our case, combination therapy resulted in prompt resolution of respiratory symptoms, normalization of inflammatory markers, and significant radiographic improvement, ultimately leading to a favorable clinical outcome.

Early identification of the causative pathogen not only facilitates targeted therapy but also minimizes unnecessary exposure to broad-spectrum antibiotics.

## Conclusion

This case highlights the diagnostic and therapeutic complexity of pulmonary nocardiosis in immunocompetent individuals, especially when clinical and radiological features mimic malignancy. The rapid identification of *Nocardia saintgeorgesii* by mNGS proved critical in achieving an accurate diagnosis and guiding effective antimicrobial therapy, thereby avoiding unnecessary invasive interventions.

Beyond the diagnostic challenges, this report draws attention to a potentially overlooked association between Nocardia infection and thromboembolic complications. The concurrent occurrence of pulmonary embolism in this patient, in the absence of classical predisposing factors, suggests that systemic inflammation and endothelial activation induced by Nocardia infection may contribute to a hypercoagulable state. This observation warrants heightened clinical vigilance and systematic evaluation for thrombotic events in patients with pulmonary nocardiosis.

Early recognition and pathogen-specific management remain the cornerstones of improving outcomes in PN. Clinicians should maintain a high index of suspicion for Nocardia infection when encountering atypical pulmonary masses unresponsive to empirical antibiotics, even in immunocompetent hosts. Future research should focus on elucidating the underlying mechanisms linking Nocardia infection with coagulation disturbances.

## Data Availability

The original contributions presented in the study are included in the article/supplementary material. Further inquiries can be directed to the corresponding authors.
